# GAS5 regulated by FTO-mediated m6A modification suppresses cell proliferation via the IGF2BP2/QKI axis in breast cancer

**DOI:** 10.1007/s12672-024-01051-8

**Published:** 2024-05-23

**Authors:** Yuzhao Yan, Jing Ma, Qingqiu Chen, Ting Zhang, Rui Fan, Junze Du

**Affiliations:** 1 Department of Breast and Thyroid Surgery, Southwest Hospital, Army Medical University, Chongqing, 400038 China; 2 Key Laboratory of Chongqing Health Commission for Minimally Invasive and Precise Diagnosis and Treatment of Breast Cancer, Chongqing, 400038 China; 3 Department of Radiology, Southwest Hospital, Army Medical University, Chongqing, 400038 China

**Keywords:** LncRNA GAS5, FTO, IGF2BP2, QKI, Breast cancer

## Abstract

**Background:**

The lncRNA growth arrest-specific 5 (GAS5) is involved in regulating breast cancer progression. In this study, we aimed to elucidate the function and mechanism of GAS5 in breast cancer.

**Methods:**

The expressions of GAS5, fat mass and obesity-associated protein (FTO), insulin-like growth factor 2 mRNA-binding protein 2 (IGF2BP2), and Quaking (QKI) were assessed by quantitative reverse transcription-polymerase chain reaction (qRT-PCR) and western blot. The m6A modification level of GAS5 was detected using m6A immunoprecipitation assay (MeRIP). The interaction between IGF2BP2 and GAS5 or QKI was detected using RNA immunoprecipitation assay (RIP) and dual luciferase reporter assay. Cell proliferation was measured using the Cell Counting Kit-8 (CCK-8) assay. The biological functions of the FTO/GAS5/IGF2BP2/QKI axis was assessed using the tumor xenograft assay.

**Results:**

LncRNA GAS5 expression decreased in breast cancer and was regulated by FTO-mediated m6A modification in an IGF2BP2-dependent manner, resulting in decreased GAS5 stability and expression. GAS5 recruited IGF2BP2 to target QKI and upregulated QKI expression in breast cancer cells. GAS5 suppressed breast cancer growth via IGF2BP2/QKI, and this inhibitory effect was modulated by FTO both in vitro and in vivo.

**Conclusions:**

GAS5 regulated by FTO-mediated m6A modification represses the growth of breast cancer via the IGF2BP2/QKI pathway, suggesting that the FTO/GAS5/IGF2BP2/QKI pathway can be a potential target for breast cancer treatment.

## Introduction

According to statistics, breast cancer is a common malignant tumor among women in the world, and the incidence of breast cancer in women is increasing each year [1]. Novel advanced therapeutic strategies including targeted therapy, endocrine therapy, and immunotherapy have been applied in clinical treatment, and improved the survival rates for breast cancer patients. However, the prognosis of breast cancer is still unsatisfactory. Therefore, it is necessary to further study the molecular mechanism of breast cancer progression and explore new targets for breast cancer treatment.

Long non-coding RNAs (lncRNAs) are a group of endogenous non-coding RNAs longer than 200 nucleotides. LncRNAs currently are considered as an important regulatory molecule of various tumor progression through a variety of molecular mechanisms. The lncRNA GAS5 is downregulated in multiple cancers and acts as a tumor suppressor, including gastric cancer, lung cancer, hepatocellular carcinoma, cervical cancer, prostate cancer, and glioma [2, 3]. In breast cancer, low GAS5 expression is associated with clinical characteristics [4–7], and restrains breast cancer progression. However, the mechanism of GAS5 dysregulation is still unclear and molecular functions of GAS5 still need further study.

N6-methyladenosine (m6A) modification, methylated at the N6 position of adenosine, is the most abundant internal epi-transcriptomic modification of eukaryotic mRNAs and non-coding RNAs [8, 9]. m6A modification plays a critical role in regulating RNA transcription, processing, translation, and degradation [10–12], which is dynamically regulated by m6A methyltransferase (METTL3, METTL14 and WTAP) [13, 14], demethylase (FTO and ALKBH5) [15, 16], and m6A-binding proteins (YTH domain family proteins, HNRNPA2B1 and IGF2BP) [17–20]. FTO protein, originally known to be associated with increased body mass and obesity in children and adults [21], is the first identified m6A demethylase to perform m6A demethylation activity on mRNAs [17]. Recent studies have demonstrated that FTO acts as an oncogene to promote the progression of various human cancers including breast cancer [22–25], suggesting that FTO may be a potential target for cancer therapy. However, the underlying mechanism of FTO as a demethylase in breast cancer progression still needs further investigation.

In the present study, we found that the expression of lncRNA GAS5 was decreased in breast cancer and regulated by FTO-mediated m6A modification. Next, we demonstrated that FTO downregulated GAS5 expression in an IGF2BP2-dependent manner. GAS5 recruited IGF2BP2 to target QKI and upregulated QKI expression in breast cancer cells. Furthermore, biological function experiments showed that GAS5 suppressed breast cancer growth via IGF2BP2/QKI, and this inhibitory effect was modulated by FTO both in vitro and in vivo, thus representing a promising strategy for the treatment of breast cancer.

## Materials and methods

### Tissue samples

Thirty paired breast cancer and adjacent noncancerous tissue samples were collected from patients undergoing breast cancer surgery in Department of Breast and Thyroid Surgery, Southwest Hospital, Army Medical University. Before surgery, none of the patients had undergone radiotherapy, chemotherapy, or other special treatments. The surgically removed fresh tissues were immediately put into liquid nitrogen to avoid RNA degradation. The detailed clinical information of all patients is shown in Table 1. The application of patient-derived materials and the protocols were approved by the Ethics Committee of the First Affiliated Hospital of Army Medical University (No: KY2023070) in accordance with the Declaration of Helsinki or comparable ethical standards.

**Table 1:** Clinical characteristics of breast cancer patients

Characteristics	Patients (n)	%
Age (years)		
≤ 45	7	23.3
46–60	17	57.7
≥ 61	6	20.0
TNM stage		
I-II	25	83.3
III-IV	5	16.7
ER status		
Negative	3	10.0
Positive	27	90.0
PR status		
Negative	5	16.7
Positive	25	83.3
HER2 status		
Negative	28	93.3
Positive	2	6.7
TNBC		
Yes	3	10.0
No	27	90.0

*ER* estrogen receptor; *PR* progesterone receptor; *HER2* human epidermal growth factor receptor 2; TNBC *ER*^*−*^*/PR*^*−*^*/ HER2*^*−*^;

### Cell culture

The human breast cancer cell lines BT-474, SKBR3, BT-20, MDA-MB-231, MCF-7, and T-47D were obtained from American Type Culture Collection (ATCC). Cells were cultured in Dulbecco’s modified eagle medium (DMEM) (Gibco, USA) supplemented with 10% fetal bovine serum (FBS) (Gibco, USA) and 1% streptomycin-penicillin in a humidified atmosphere at 37 °C with 5% CO_2_.The immortalized mammary epithelial cell line MCF10A were obtained from ATCC and cells were cultured in Dulbecco’s Modified Eagle Medium: F-12(DMEM/F-12) (Gibco, USA) supplemented with 5% horse serum, 50 ng/ml epidermal growth factor (EGF), 10 mg/ml insulin, 0.5 mg/ml hydrocortisone, 0.1 mg/ml cholera toxin and 1% streptomycin-penicillin at 37 °C in an atmosphere of 5% CO2 and 95% air.

### Plasmid construction and cell transfection

The full-length fragments of GAS5, FTO, and IGF2BP2 were synthesized by Sangon Biotech Co., Ltd. (Shanghai, China) and subcloned into the pcDNA3.1 vector, named GAS5-OE, FTO-OE, and IGF2BP2-OE. Using the SRAMP database (http://www.cuilab.cn/sramp), we predicted the potential m6A modification sites (DRACH) in GAS5 mRNA. According to the predicted m6A modification sites, which were located at GAS5 mRNA position 82(GGACA), 274(GGACA), and 362(GGACT), the dual-luciferase reporter containing fragments of m6A modification sites (wild-type) were synthesized and subcloned into the pmirGlo luciferase expression vector by Sangon Biotech Co., Ltd. (Shanghai, China). We mutated the predicted m6A binding site, and the mutation sequence is as follows: mutant position 82(GGACA), mutant position 274(GGACA), and mutant position 362(GGACT). Then, the fragment containing mutations in m6A binding sites were also synthesized and cloned into the pmirGlo luciferase expression vector by Sangon Biotech Co., Ltd. (Shanghai, China), and the resulting recombinant plasmids were termed pmir-GAS5-WT and pmir-GAS5-Mut, respectively. Next, cells were transfected with siRNA or plasmids using Lipofectamine 3000 transfection reagent (ThermoFisher, DE, USA) according to the manufacturer’s instructions, followed by test of the cells accordingly.

### Quantitative reverse transcription-polymerase chain reaction (qRT-PCR)

Total RNA was extracted from tissues or cells using Trizol reagent (ThermoFisher, USA) according to the manufacturer’s protocol. Complementary DNA was synthesized using PrimeScript RT reagent kit (Takara, Dalian, China). SYBR Premix Ex Taq II (Takara, Dalian, China) was applied to performing qPCR. The qPCR primers were: GAS5 forward primer, 5′- ACTCCTGTGAGGTATGGTGC -3′, and reverse primer, 5′- TGTCTAATGCCTGTGTGCCAA -3′; FTO forward primer, 5′-ACTTGGCTCCCTTATCTGACC-3′, and reverse primer, 5′- TG TG CA GTGTGAGAAAGGCTT -3′; IGF2BP2 forward primer, 5′-GTCCTGGACACTACC ACGTT-3′ and reverse primer, 5′-TCCATCCAACACCTCCCACT-3′; QKI forward primer, 5′-GGCTTTCTAAATCCAGGGAGCA -3′, and reverse primer, 5′-GTTGACAACGGCGGTTTCTG-3′; β-actin forward primer, 5′- AGCGAGCATCCCCCAAAGTT-3′, and reverse primer, 5′- GGGCACGAAGGCTCATCATT-3′. The relative mRNA expression level was analyzed using the 2^−ΔΔCt^ method, taking β-actin as an endogenous control.

### Western blot

Whole proteins were extracted with RIPA and the concentrations of protein were determined via a BCA kit (Beyotime, Shanghai, China). The protein samples in each group were separated by 10% sodium dodecyl sulphate–polyacrylamide gel electrophoresis (SDS-PGAE), followed by transferring onto polyvinylidene difluoride (PVDF) membranes (Millipore, MA, USA). Then, membranes were incubated with antibodies against FTO (ab280081,1:1000, Abcam), IGF2BP2 (ab124930,1:1000, Abcam), QKI (ab126742,1:1000, Abcam) and β-actin (ab8227,1:1000, Abcam) at 4 °C overnight. Next, the membranes were incubated with the corresponding secondary antibody (ab7090, 1:10000, Abcam) at 37 °C for 1 h. After washing, protein bands were visualized with enhanced chemiluminescence detection reagent (Thermo Fisher Scientific) and imaged by ChemiDoc™ Touch imaging system (Bio-Rad, CA, USA).

### RNA stability analysis

Transfected cells were treated with 5 μg/mL of actinomycin D (sigma, USA). At different time points after actinomycin D treatment, cells were harvested to extract total RNA, and the relative RNA levels were detected by qRT-PCR assay.

### m6A-RNA immunoprecipitation (MeRIP)

The m6A immunoprecipitation (MeRIP) assay was performed using an m6A-methylated RNA Immunoprecipitation Kit (Merck Millipore, MA, USA) according to the manufacturer’s instructions. Briefly, RNA was extracted and fragmented into ∼100 nt. Then the fragmented RNA was incubated with magnetic beads pre-conjugated with m6A antibody or IgG antibody. Next, the m6A-enriched RNAs were extracted and analyzed by qPCR assay.

### RNA immunoprecipitation (RIP) assay

RNA immunoprecipitation (RIP) assay was performed using the Magna RIP RNA-Binding Protein Immunoprecipitation Kit (Merck Millipore, MA, USA). Cell lysate was incubated with magnetic beads preincubated with IGF2BP2 antibody or IgG antibody. The co-precipitated RNAs were extracted and examined by RT-qPCR assay.

### Dual luciferase reporter assay

Wild-type or mutant report plasmids containing m6A modification sites were co-transfected with IGF2BP2 overexpression plasmids into breast cancer cells using Lipofectamine 3000 reagent (ThermoFisher, USA). The dual-luciferase reporter assay kit (Promega, WI, USA) was used to measure the luciferase activities according to the manufacturer’s instructions, and the renilla luciferase activity was measured as an internal control.

### Cell proliferation assay

Breast cancer cells were transfected with plasmid or siRNA for 24 h using lipofectamine 3000 reagent. Then, cells (5000 cells/well) were seeded into 96-well plates cultured for different time periods, followed by adding of Cell Counting Kit-8 (CCK-8) reagent (Dojindo, Shanghai, China) into each well and incubation for 4 h at 37 °C. The optical density (OD) of cell solution was determined with a spectrophotometer at 450 nm.

### Tumor xenograft assay

Female BALB/c nude mice (4 weeks old) were purchased from the Vital River Laboratory Animal Technology (Beijing, China). Animal caring and experiments were approved by the Ethics Committee of the First Affiliated Hospital of Army Medical University (No: AMUWEC20232903), and carried out in compliance with the ARRIVE guidelines. Briefly, 1 × 10^7^ MCF-7 cells with GAS5, IGF2BP2, QKI, and FTO stably expressed or silenced were subcutaneously injected into the right axilla of each nude mouse. Then the tumor growth was recorded and the tumor volumes were measured using the following formula: volume = width^2^ × length × 1/2. After four weeks, all the mice were sacrificed by cervical dislocation, and the xenograft tumors were resected and photographed. The expressions of the protein and RNA in tumor tissues were detected by western blot and qPCR.

### Statistical analysis

GraphPad Prism software version 9.0 was used for statistical analyses. Data are expressed as mean ± standard deviation (SD) from three independent experiments. The differences were determined using the Student’s t-test. *P* < 0.05 was considered statistically significant.

## Results

### GAS5 was downregulated in breast *cancer* and negatively correlated with FTO expression

**Fig. 1:**
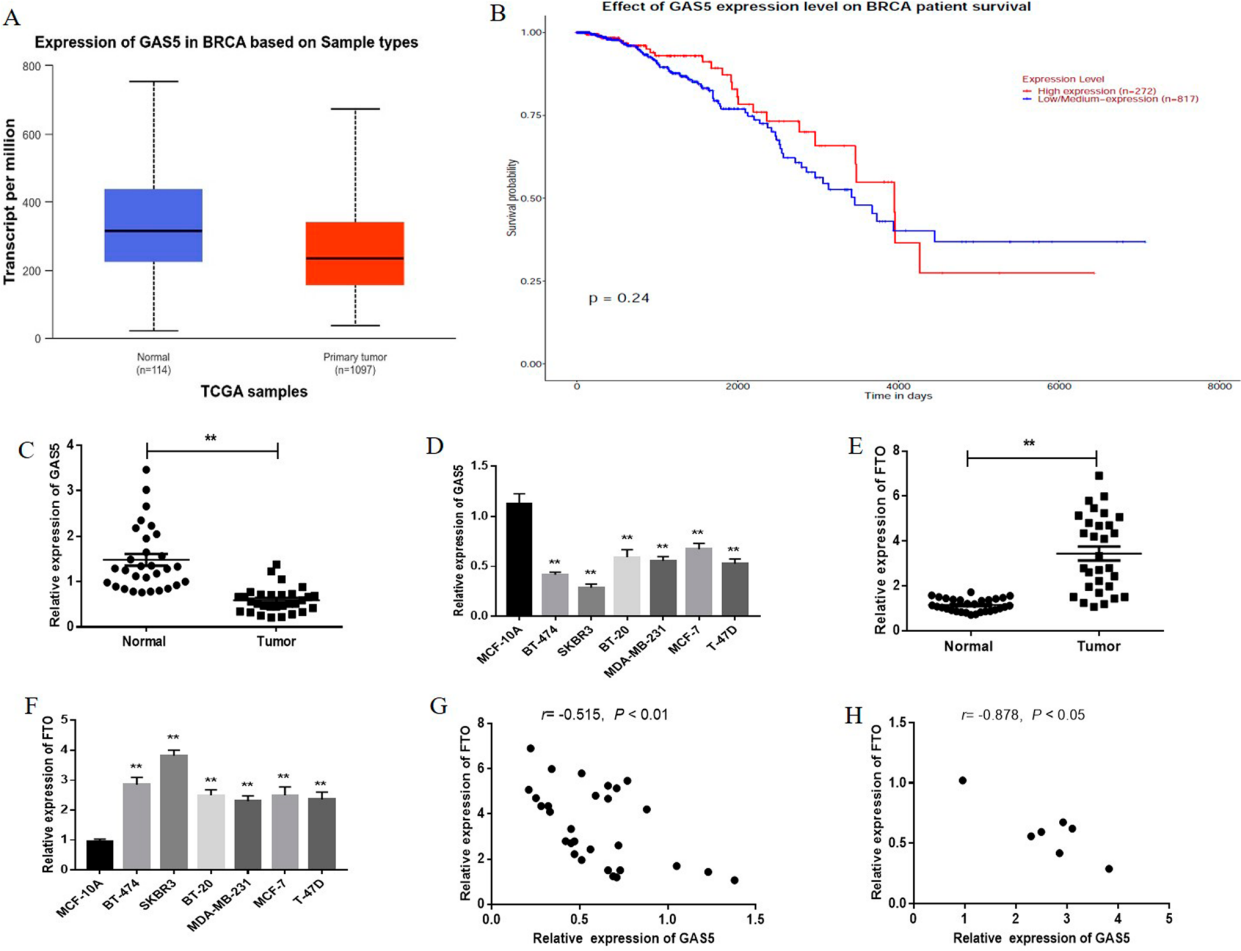
GAS5 is downregulated in breast cancer and negatively correlated with FTO expression. **A** GAS expression in breast cancer was analyzed according to the UALCAN database. **B** The overall survival (OS) curves of breast cancer with high or low expression of GAS5 was from GEPIA database. **C** The levels of GAS5 was detected by qRT-PCR in 30 samples of breast cancer tissue and adjacent non-tumor normal tissue. **D** The expression of GAS5 in breast cancer cell lines and human breast epithelial cell line MCF-10A was assayed by qRT-PCR. **E** FTO expression in 30 breast cancer and adjacent non-tumor tissue samples was detected by qRT-PCR. **F** FTO expression in breast cancer cell lines and human breast epithelial cell line MCF-10A was detected by qRT-PCR. **G** Correlation between GAS5 and FTO expression in breast cancer tissues was analyzed by Pearson’s correlation coefficient. **H** The correlation between the levels of GAS5 and FTO in breast cancer cell lines was analyzed using Pearson’s test. Data are shown as mean ± SD. *, *P* < 0.05; **, *P* < 0.01

We first analyzed GAS5 expression in breast cancer through UALCAN database, and found that GAS5 was downregulated in breast cancer tissues compared with that in normal tissues (Fig. 1A). Kaplan–Meier OS curve from UALCAN database suggested that the low expression of GAS5 predicted poor prognosis (Fig. 1B). Also, we examined the expression of GAS5 in breast cancer tissues and in the corresponding adjacent noncancerous normal tissues by qRT-PCR. As shown in Fig. 1C, the expression of GAS5 was significantly lower in breast cancer tissues than in adjacent noncancerous normal tissues. Moreover, we found that GAS5 was more lowly expressed in breast cancer cell lines (Fig. 1D). To further explore the mechanism underlying the low expression of GAS5 in breast cancer, we focused on m6A modification, which has been reported to play an important role in the progression of multiple tumors. Recent studies have revealed that FTO, an important molecule involved in m6A modification, is closely involved in a variety of tumors including breast cancer. Recent studies suggested that FTO, an important molecule involved in m6A modification, is closely involved in a variety of tumors including breast cancer [22–25]. Therefore, we assumed that there could be a possible correlation between GAS5 and FTO. Then, we examined the expression of FTO in breast cancer tissues and cells. As shown in Fig. 1, the expression of FTO was relatively higher in breast cancer tissues (Fig. 1E) and cells (Fig. 1F). Moreover, there was a negative correlation between the expression of GAS5 and FTO in breast cancer tissues and cells (Fig. 1G and H). These results suggest that the downregulation of GAS5 in breast cancer is probably associated with FTO.

### GAS5 was downregulated by FTO-mediated m6A modification

**Fig. 2:**
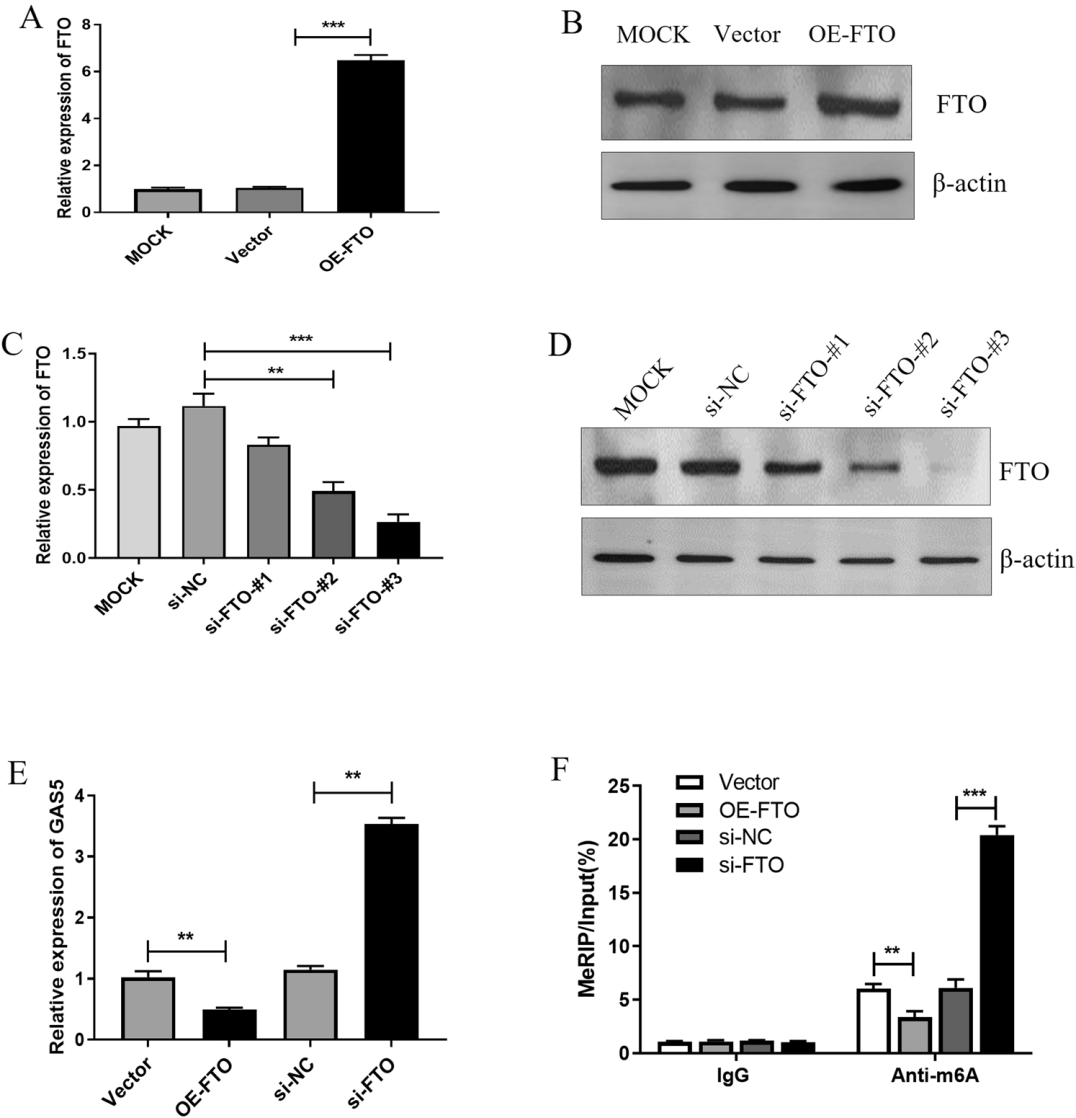
GAS5 expression is downregulated by FTO-mediated m6A modification. **A**, **B** MCF-7 cells were transfected with transfection reagent (Mock), Vector, OE-FTO for 24 h, and then the mRNA (**A**) and protein (**B**) levels of FTO were measured by qRT-PCR and western blot. **C**, **D** MCF-7 cells were transfected with Mock, si-NC (negative control siRNA) or si-FTO (#1, #2, and #3) for 24 h, and then the mRNA (**C**) and protein (**D**) levels of FTO were measured by qRT-PCR and western blot. **E** MCF-7 cells were transfected with vector, OE-FTO, si-NC or si-FTO for 24 h, and then the level of GAS5 was assayed by qRT-PCR. **F** GAS5 m6A levels in MCF-7 cells were analyzed by MeRIP-qPCR assay. Data are presented as mean ± SD. **, *P* < 0.01; ***,* P* < 0.001

To further investigate the regulatory effect of FTO on GAS5, overexpression (Fig. 2A and B) or knockdown of FTO (Fig. 2C and D) was performed in MCF-7 cells. We selected si-FTO #3 for subsequent experiments due to its most significant inhibitory effect on FTO. As shown in Fig. 2, FTO overexpression decreased the GAS5 level, whereas FTO knockdown increased the GAS5 level in MCF-7 cells (Fig. 2E). Furthermore, we observed that the m6A modification of GAS5 was enhanced by FTO knockdown while reduced by FTO overexpression (Fig. 2F). Overall, these results suggest that FTO decreases GAS5 expression by regulating the m6A demethylation of GAS5.

### FTO downregulated GAS5 expression in an IGF2BP2-dependent manner

**Fig. 3:**
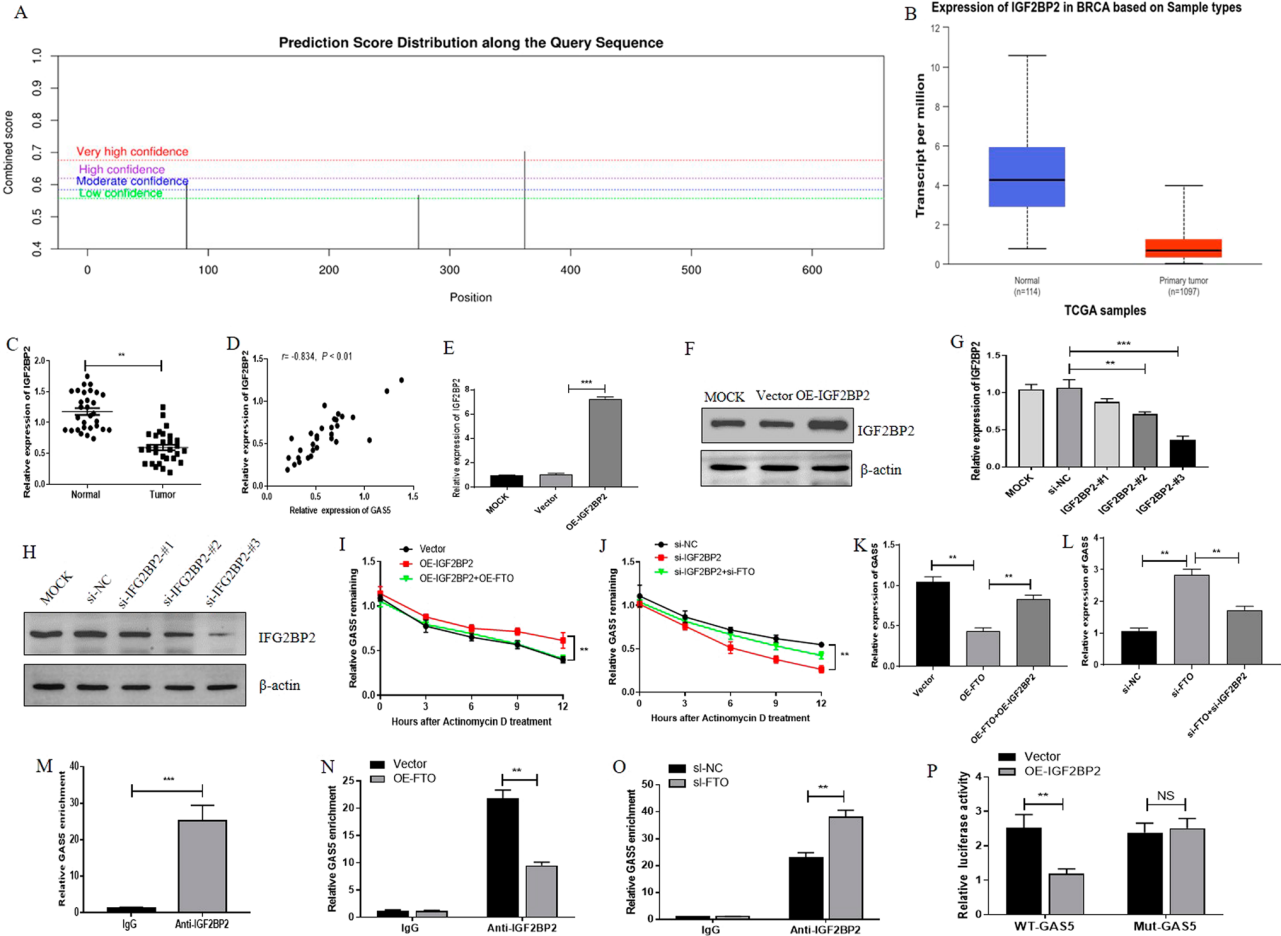
FTO downregulates GAS5 expression in an IGF2BP2-dependent manner. **A** The potential m6A modification sites in GAS5 were analyzed using the SRAMP database. **B** IGF2BP2 expression in breast cancer was analyzed according to the UALCAN database.** C** IGF2BP2 expression was detected by qRT-PCR in 30 samples of breast cancer tissue and adjacent non-tumor tissue. **D** The correlation between GAS5 and IGF2BP2 expression in breast cancer tissues was analyzed by Pearson’s correlation coefficient. **E**, **F** MCF-7 cells were transfected with Mock, Vector, or OE-FTO for 24 h, and then the IGF2BP2 mRNA (**E**) and protein (**F**) levels were measured by qRT-PCR and western blot. **G**, **H** MCF-7 cells were transfected with Mock, si-NC or si- IGF2BP2 (#1, #2, and #3) for 24 h, and then the IGF2BP2 mRNA (**G**) and protein (**H**) levels were measured by qRT-PCR and western blot. **I, J** After 48 h post-transfection, the GAS5 stability was detected by RNA stability analysis. **K** Cells were transfected with vector, OE-IGF2BP2, or OE-FTO for 24 h, and then the level of GAS5 was assayed by qRT-PCR. **L** Cells were transfected with si-NC, si-FTO, or si-IGF2BP2 for 24 h, and then the level of GAS5 was assayed by qRT-PCR. **M** The level of GAS5 enriched by IgG or anti-IGF2BP2 was examined by RIP assay. **N**, **O** Cells were transfected with vector, OE-FTO, si-NC or si-IGF2BP2, and then the effect of FTO on the combination of IGF2BP2 and GAS5 was validated by RIP assay. **P** MCF-7 cells were co-transfected with 0.5 μg of indicated dual-luciferase reporter (pmir-GAS5-WT and pmir-GAS5-Mut, respectively), and 0.5 μg of OE-IGF2BP2 or vector, and the relative luciferase activity was measured. The firefly luciferase activity was normalized to Renilla luciferase activity. **, *P* < 0.01; ***,* P* < 0.001; NS, not significant

It has been well documented that m6A readers can recognize m6A-modified RNAs to influence their localization, stability and translation. Among the m6A reader proteins, IGF2BP2 has been reported to be closely involved in tumor progression by binding to m6A modification sites to regulate the stability and translation of target mRNAs [20, 26]. Using the online database SRAMP (http://www.cuilab.cn/sramp), we predicted several potential m6A modification sites in GAS5, suggesting that IGF2BP2 may recognize and bind to methylated GAS5 (Fig. 3A). We also found decreased IGF2BP2 expression in breast cancer tissues through UALCAN database (Fig. 3B). In addition, detection of IGF2BP2 expression in breast cancer tissues and corresponding adjacent noncancerous normal tissues revealed the decrease of IGF2BP2 in breast cancer tissues (Fig. 3C). Correlation analysis showed that the expression of IGF2BP2 was positively correlated with that of GAS5 (Fig. 3D). Based on the above results, we speculated that IGF2BP2 might mediate the regulatory effect of FTO on GAS5. Thus, we first overexpressed (Fig. 3E and F) and silenced IGF2BP2 in breast cancer cells (Fig. 3G and H), and si-IGF2BP2-#3 with the best inhibitory effect was selected for the follow-up experiments. Accordingly, we found that ectopic IGF2BP2 expression enhanced GAS5 stability while silencing IGF2BP2 decreased GAS5 stability, which could be restored by FTO abundance or deficiency, respectively (Fig. 3I and J). In addition, overexpression of IGF2BP2 weakened the inhibitory effect of FTO abundance on the GAS5 level (Fig. 3K), whereas IGF2BP2 silence attenuated the elevation of GAS5 expression caused by FTO silence (Fig. 3L). Furthermore, using the online database StarBase (http://starbase.sysu.edu.cn/), we predicted that IGF2BP2 might bind to GAS5. Then, the RIP assay confirmed that GAS5 combined with IGF2BP2 (Fig. 3M), and the combination of IGF2BP2 with GAS5 was regulated by knockdown or overexpression of FTO (Fig. 3N and O). Moreover, the luciferase activity of wild-type (WT) GAS5 reporter (containing WT m6A binding sites) was markedly reduced by IGF2BP2 overexpression, but this effect was abolished by a mutant-type (MUT) GAS5 reporter (Fig. 3P). Taken together, these results suggested that FTO caused GAS5 down-regulation in an IGF2BP2-dependent manner.

### GAS5 recruited IGF2BP2 to target QKI and upregulated its expression in breast *cancer* cells

**Fig. 4:**
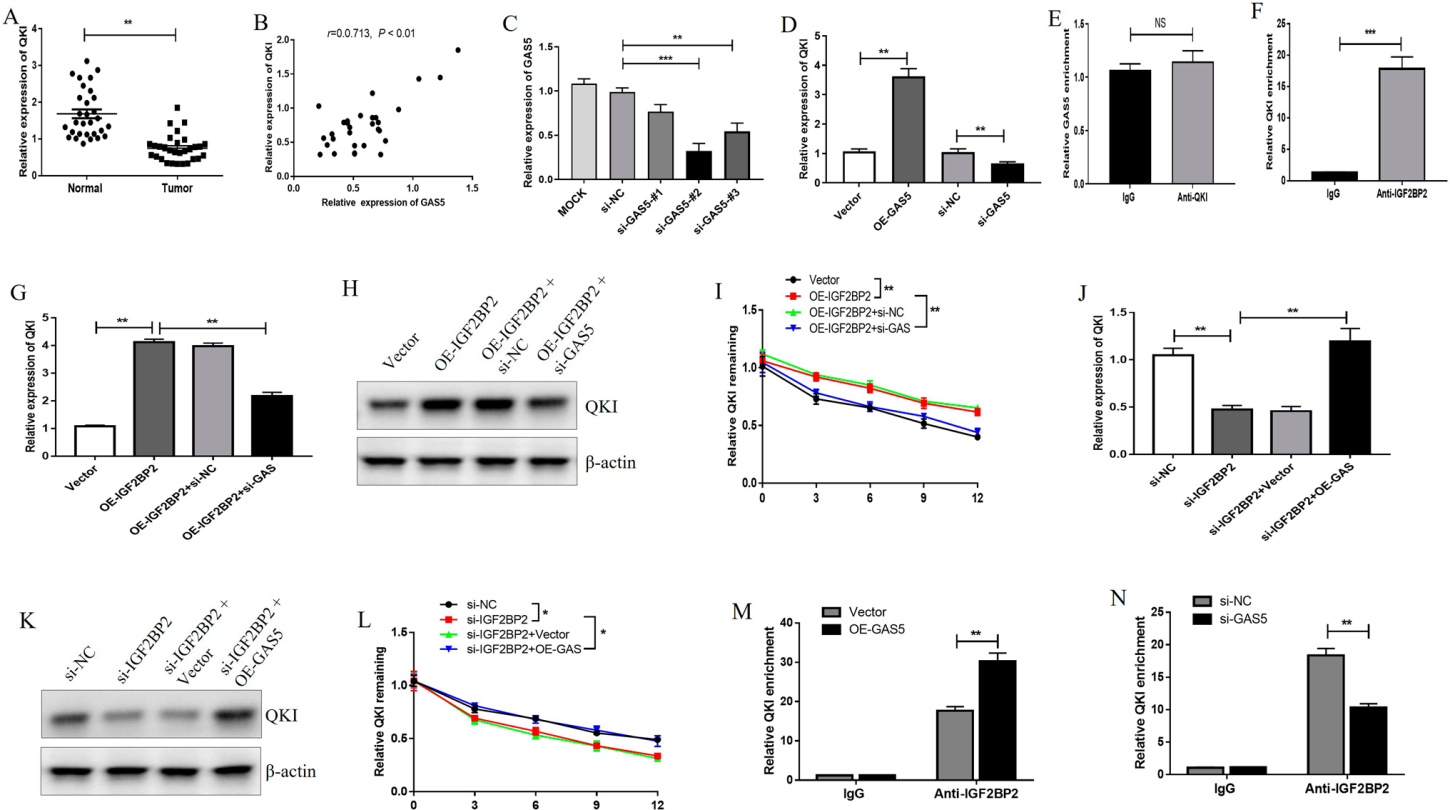
GAS5 promotes the expression of QKI by recruiting IGF2BP2. **A** The level of QKI was detected by qRT-PCR in 30 samples of breast cancer tissue and adjacent non-tumor normal tissue. **B** The correlation between GAS5 and QKI expression in breast cancer tissues was analyzed by Pearson’s correlation coefficient. **C** MCF-7 cells were transfected with Mock, vector, OE-GAS5, si-NC or si-GAS5 (#1, #2, and #3) for 24 h, and then the GAS5 expression was detected by RT-qPCR. **D** Cells were transfected with vector, OE-GAS5, si-NC or si-GAS5, and then the QKI expression was detected by RT-qPCR. **E**, **F** The combination of QKI with GAS5 or IGF2BP2 was verified by RIP. **G**–**I** Cells were transfected with vector, OE-IGF2BP2, si-NC or si-GAS5, and then the QKI mRNA (**G**) and protein (**H**) levels were measured by qRT-PCR and western blot, and the QKI stability was detected by RNA stability analysis (**I**). **J-L** Cells were transfected with vector, OE-GAS5, si-NC or si-IGF2BP2, and then the QKI mRNA (**J**) and protein (**K**) levels were measured by qRT-PCR and western blot, and the QKI stability was detected by RNA stability analysis (**L**). **M**, **N** Cells were transfected with vector, OE-GAS5, si-NC or si-GAS5, and then the effect of GAS5 on the combination of IGF2BP2 with QKI was examined by RIP assay. *P < 0.05, **P < 0.01, ***P < 0.001, NS, not significant

To further explore the molecular mechanism of GAS5, we predicted the downstream targets of GAS5 using the StarBase database. QKI, a tumor suppressor that represses breast cancer, was predicted as a possible target of GAS5. We also found that QKI expression was low in breast cancer tissues and positively correlated with GAS5 expression (Fig. 4A and B), suggesting that GAS5 may regulate QKI. To further explore the regulatory effect of GAS5 on QKI and related mechanisms, GAS5 was overexpressed and knocked down (si-GAS5 #2 was used for follow-up experiments owing to its best inhibitory effect) (Fig. 4C). As shown in Fig. 4, QKI expression was significantly enhanced after GAS5 overexpression while decreased after GAS5 deficiency (Fig. 4D), indicating that GAS5 may affect QKI expression. However, we did not observe the direct binding of GAS5 to QKI by RIP assay (Fig. 4E), suggesting that GAS5 may indirectly regulate QKI. In view of the above RIP assay results (Fig. 4E), and our prediction of the potential binding of IGF2BP2 to QKI through StarBase database, we speculated that GAS5 may indirectly regulate QKI through recruiting IGF2BP2. As expected, the RIP assay verified the interaction between IGF2BP2 and QKI (Fig. 4F). Moreover, overexpression of IGF2BP2 increased the expression and stability of QKI (Fig. 4G-I), but IGF2BP2 reduction decreased the expression and stability of QKI (Fig. 4J-L), which was reversed by GAS5 knockdown and overexpression, respectively (Fig. 4G-L). Furthermore, we found that GAS5 could affect the combination of IGF2BP2 with QKI significantly (Fig. 4M and N). In sum, these results demonstrated that GAS5 recruited IGF2BP2 able to target QKI to enhance the stability expression of QKI.

### GAS5 suppressed breast *cancer* growth via IGF2BP2/QKI and the suppression was regulated by FTO in vitro and in vivo

**Fig. 5:**
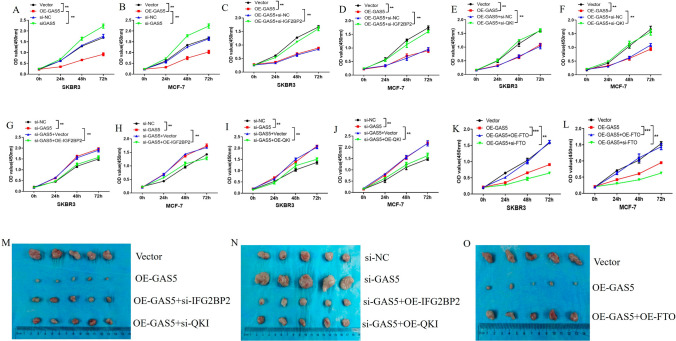
GAS5 suppresses breast cancer growth via IGF2BP2/QKI and the inhibition was regulated by FTO in vitro and in vivo.** A**, **B** SKBR3 and MCF-7 cells were transfected with vector, OE-GAS5, si-NC, or si-GAS5, and then the cell proliferation capacities were examined by CCK-8 assay. **C**–**F** Cells were transfected with vector, OE-GAS5, si-NC, si-IGF2BP2 or si-QKI, and then the cell proliferation capacities were examined by CCK-8 assay. **G**-**J** Cells were transfected with si-NC, si-GAS5, vector, OE-IGF2BP2, or OE-QKI, and then the cell proliferation capacities were examined by CCK-8 assay. **K**, **L** SKBR3 and MCF-7 cells were transfected with vector, OE-GAS5 + OE-FTO, or OE-GAS5 + si-FTO, and then the role of FTO in regulating the inhibitory effect of GAS5 on cell proliferation was evaluated by CCK8 assay. **M**–**O** Xenograft assays were used to assess the role of the FTO/GAS5/IGF2BP2/QKI axis. *, *P* < 0.05; **, *P* < 0.01

Based on the above results, GAS5 was downregulated by the m6A demethylase FTO, and GAS5 can upregulate QKI by recruiting IGF2BP2. Next, we explored the biological functions of the FTO/GAS5/IGF2BP2/QKI axis in breast cancer. As shown in Fig. 5, overexpression of GAS5 inhibited the proliferation of breast cancer cells, while knockdown of GAS5 promoted the proliferation of breast cancer cells (Fig. 5A and B). In addition, knockdown or overexpression of IGF2BP2 or QKI reversed the effect of GAS5 on the proliferation of breast cancer cells, respectively (Fig. 5C–J). Moreover, overexpression or knockdown of FTO affected the inhibitory effect of GAS5 on the proliferation of breast cancer cells (Fig. 5K and L). Accordingly, xenograft assay showed that GAS5 overexpression attenuated tumor growth, while GAS5 silencing promoted tumor growth in vivo, which could be reversed by knockdown or overexpression of IGF2BP2 or QKI (Fig. 5M and N). Furthermore, overexpression of FTO attenuated the inhibitory effect of GAS5 on tumor growth in vivo (Fig. 5O). These results suggested that GAS5 suppressed breast cancer growth via IGF2BP2/QKI, and this inhibitory effect was modulated by FTO both in vitro and in vivo.

## Discussion

Once being considered functionless, lncRNAs are now recognized to play an important role in various physiological and biochemical processes, including regulating the expression of key genes. Accumulating studies have confirmed that lncRNAs are closely involved in the occurrence and development of different tumor types, and a large number of lncRNAs have become biomarkers of cancer diagnosis and prognosis. Some lncRNAs act as oncogenes to promote tumor, while others serve as tumor suppressor genes to inhibit tumor [27–29]. LncRNA GAS5, which is lowly expressed in most cancers, can suppress the expression of many oncogenes, and also activate the expression of other tumor suppressor genes [2, 30]. Previous reports have confirmed that low expression of GAS5 is associated with tumor recurrence, metastasis, low survival rate, and chemotherapy tolerance [31]. Consistent with previous studies including those on breast cancer, we found that GAS5 was underexpressed in breast cancer tissues and cells.

It is well established that m6A modification is a dynamic process, which regulates various aspects of RNA expression, including mRNA stability, splicing, translation efficiency, nuclear export, and degradation [32]. In recent years, the role of m6A modification in cancer including breast cancer receives increasing attention. Previous reports showed that the upregulation of FTO enhanced cell migration and invasion by affecting the miR-181b-3p (microRNAs, miRNAs)/ARL5B signaling pathway in breast cancer [23]. FTO mediated the m6A demethylation of BNIP3 mRNA and induced its degradation, thus promoting breast tumor progression [24]. FTO promotes the stability and expression of ZEB1 transcripts by decreasing m6A RNA methylation, leading to chemoresistance and epithelial-mesenchymal transition (EMT) of breast cancer cells [33]. FTO overexpression enhanced the doxorubicin resistance of breast cancer cells by STAT3 pathway [34]. Consistent with previous reports, we showed that FTO expression was relatively higher in breast cancer tissues and cells. In addition, there was a negative correlation between the expression of GAS5 and FTO. Our results also revealed that FTO overexpression decreased GAS5 level and the stability by regulating the m6A modification of GAS5.

GAS5 has been proven to inhibit breast cancer as a tumor suppressor through a variety of functions and mechanisms. Prior studies have demonstrated that GAS5 inhibits breast cancer progression through adsorbing distinct miRNAs (e.g., miR-221-3p, miR-196a-5p, miR-378a-5p, miR-23a, and miR-103) as an miRNA sponge and further up-regulating miRNA target proteins [35–38]. In addition, reports have shown that GAS5 interacts with some proteins, such as E2F1, E2F4, YBX1, EZH2, and YAP, which inhibit tumor progression [39–42]. In the present study, we found that GAS5 suppressed the growth of breast cancer cells by interacting with IGF2BP2.

IGF2BP2 is an RNA-binding proteins that is involved in regulating multiple biological processes and associated with a variety of diseases and cancers. As an m6A reader, IGF2BP2 is involved in regulating m6A modification by binding to several RNAs including mRNAs, miRNAs, and lncRNAs, thereby affecting localization, stability and translation of RNAs [26]. In our present study, we revealed that GAS5 stability and expression were reduced by FTO in an IGF2BP2-dependent manner.

QKI is an RNA binding protein that acts as a tumor suppressor in multiple tumors, including colon, lung, oral, prostate and breast cancers [43, 44]. A former report has discovered that QKI significantly inhibits cell proliferation and arrests cell cycle at G1 phase via the RASA1/MAPK signaling pathway in breast cancer [45]. QKI also regulates the alternative splicing of macroH2A1 pre-mRNA, thereby inhibiting tumor cell proliferation [46]. In the present study, we demonstrated that GAS5 increased the stability and promoted the expression of QKI by recruiting IGF2BP2. Finally, we investigated the biological functions of the FTO/GAS5/IGF2BP2/QKI axis in breast cancer. We elucidated that GAS5 suppressed breast cancer growth via IGF2BP2/QKI, and this inhibitory effect could be attenuated by FTO.

In conclusion, GAS5 represses the growth of breast cancer through regulating the IGF2BP2/QKI pathway, and this inhibitory effect is modulated by FTO-mediated m6A modification, suggesting that the FTO/GAS5/IGF2BP2/QKI pathway may be a potential target for breast cancer treatment.

## Data Availability

Data are provided within the manuscript or supplementary information files.
